# The transcriptome signature analysis of the epithelial-mesenchymal transition and immune cell infiltration in colon adenocarcinoma

**DOI:** 10.1038/s41598-023-45792-y

**Published:** 2023-10-26

**Authors:** Yusri Dwi Heryanto, Seiya Imoto

**Affiliations:** 1 Division of Health Medical Intelligence, Human Genome Center, Institute of Medical Science, The University of Tokyo, 4-6-1 Shirokanedai, Minato-ku, Tokyo 108-8639 Japan; 2 Laboratory of Sequence Analysis, Human Genome Center, Institute of Medical Science, The University of Tokyo, 4-6-1 Shirokanedai, Minato-ku, Tokyo 108-8639 Japan

**Keywords:** Functional clustering, Colorectal cancer, Cancer genomics, Cancer microenvironment, Tumour immunology

## Abstract

The epithelial-mesenchymal transition (EMT) process is tightly connected to tumors’ immune microenvironment. In colon adenocarcinoma (COAD), both the EMT and immune cell infiltration contribute to tumor progression; however, several questions regarding the mechanisms governing the interaction between EMT and the immune response remain unanswered. Our study aims to investigate the cross-talk between these two processes in cases of COAD and identify the key regulators involved. We utilized the EMT and immune signatures of samples from the COAD-TCGA database to identify three subtypes of COAD: high mesenchymal, medium mesenchymal, and low mesenchymal. We observed that EMT was associated with increased tumor immune response and infiltration mediated by pro-inflammatory cytokines. However, EMT was also linked to immunosuppressive activity that involved regulatory T cells, dendritic cells, and the upregulated expression of multiple immune checkpoints, such as *PD-1, PDL-1, CTLA-4*, and others. Finally, we employed the multivariate random forest feature importance method to identify key genes, such as *DOK2* and *MSRB3*, that may play crucial roles in both EMT and the intratumoral immune response.

## Introduction

Colon cancer is the third most prevalent cancer globally and the second leading cause of cancer-related deaths^1^. As in other cancers, metastasis is an important hallmark of colon cancer^2^. Approximately 20–25% of colon cancer patients exhibit metastasis at the time of diagnosis, and 30% experience a metastatic recurrence after initial treatment^3^. The five-year relative survival rate for colon cancer with distant metastasis is 14%^4^. Metastasis is often orchestrated by the developmental process known as the epithelial-mesenchymal transition (EMT) process^5^. EMT is a cellular process in which stationary epithelial cancer cells lose their cell polarity and cell-cell adhesion, transforming into motile mesenchymal-like cells^6^. Other important enabling characteristics of colon cancer include aspects of the tumor immune microenvironment, specifically immune evasion and tumor inflammation^2^. Inflammation can promote all stages of tumorigenesis, including initiation, promotion, and metastasis^7^. Meanwhile, immune evasion is necessary for the cancer to survive^2^. EMT-related metastasis and the tumor immune process are not isolated or independent processes; instead, they intersect to influence the progression of the tumor.

Many studies have shown the close relationship between the EMT process and the COAD immune microenvironment^8–10^. Inflammation can induce EMT-transcription factors (e.g., *SNAIL, TWIST, ZEB1*) by supplying the tumor microenvironment (TME) with EMT-promoting bioactive molecules, such as *TNF*-$\alpha$, *TGF-*$\beta$, *IL6, IL8, CCL2*, and others^11^. Conversely, mesenchymal-like cancer cells can modify the TME to benefit tumor growth. Such tumor cells can attract immunosuppressive cells such as T regulatory (Treg) cells, M2 macrophages, and myeloid-derived suppressor cells (MDSC)^12–14^. Previous studies have found that EMT directly or indirectly contributes to immunosuppression^15^. EMT and the tumor immune microenvironment are known to be involved in tumors’ invasiveness and treatment resistance, independently or in collaboration^11,16^. Therefore, comprehending the mechanisms underlying the interplay between EMT and tumor immune response is essential. However, a comprehensive investigation of these mechanisms is still lacking. Understanding these mechanisms has implications for colon cancer management, including improved immunotherapies, the identification of novel biomarkers, and the development of enhanced patient stratification for personalized therapeutic approaches.

In this study, we sought to explore the EMT-immune relationship in COAD and identify its important regulators. To achieve these aims, we utilized a computational approach called transcriptomic signature analysis^17,18^ to map information from gene expression profiles into EMT and immune cells’ signatures. This method condensed information from noisy, high-dimensional transcriptomic data into relevant gene sets that are associated with EMT and immune responses. We then used these signature scores to classify the samples and compare the EMT scores to the immune cell signature scores to examine the EMT-immune relationship. Additionally, we employed multivariate random forest to select important genes involved in both the EMT and immune processes in COAD.

## Results

### Overview

**Figure 1:**
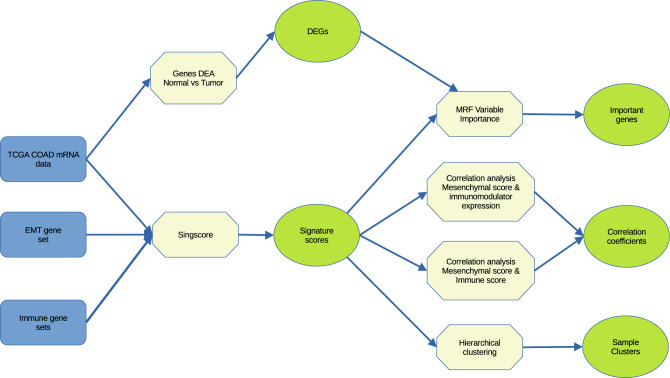
Schematic diagram of analysis steps.

Figure 1 is a schematic diagram of our methodology. We obtained the COAD dataset (consisting of normal and solid tumor samples) from the GDC-TCGA database. The COAD dataset consists of adenocarcinomas from the colon and rectosigmoid junction. Then, we conducted differential gene expression analysis on the normal and tumor samples and noted the differentially expressed genes (DEGs) for further investigation to identify crucial EMT-immune-related genes. We utilized the singscore method^19^ to map the gene expression profile of each tumor sample according to EMT and immune signatures. Then, we conducted hierarchical clustering to group the samples based on their EMT and immune signature scores, followed by a correlation analysis to explore the relationship between mesenchymal scores and immune cell scores and between mesenchymal scores and immunomodulator genes. Finally, we employed the multivariate random forest variable importance method to identify important genes in the EMT-immune relationship. We have made our analysis source codes available in the public GitHub repository (https://github.com/yusri-dh/COAD-immune-EMT) to facilitate the reproduction of our results.

### The identification of COAD clusters based on the EMT-immune signature scores

We used the EMT gene set and 39 immune-related gene sets to conduct gene set enrichment analysis on each sample, utilizing the singscore method^19^. Before conducting the enrichment analysis, we computed the gene set overlap to ensure its reliability^20^. Specifically, we calculated the Jaccard similarity between the EMT gene set and each of the 39 immune-related gene sets to measure the extent of gene set overlap. We found the highest overlap between the EMT and CSF1 response gene sets, with a Jaccard similarity of 0.031. The low Jaccard similarity across the gene sets utilized in this study affirms the robustness of our analysis.

**Figure 2:**
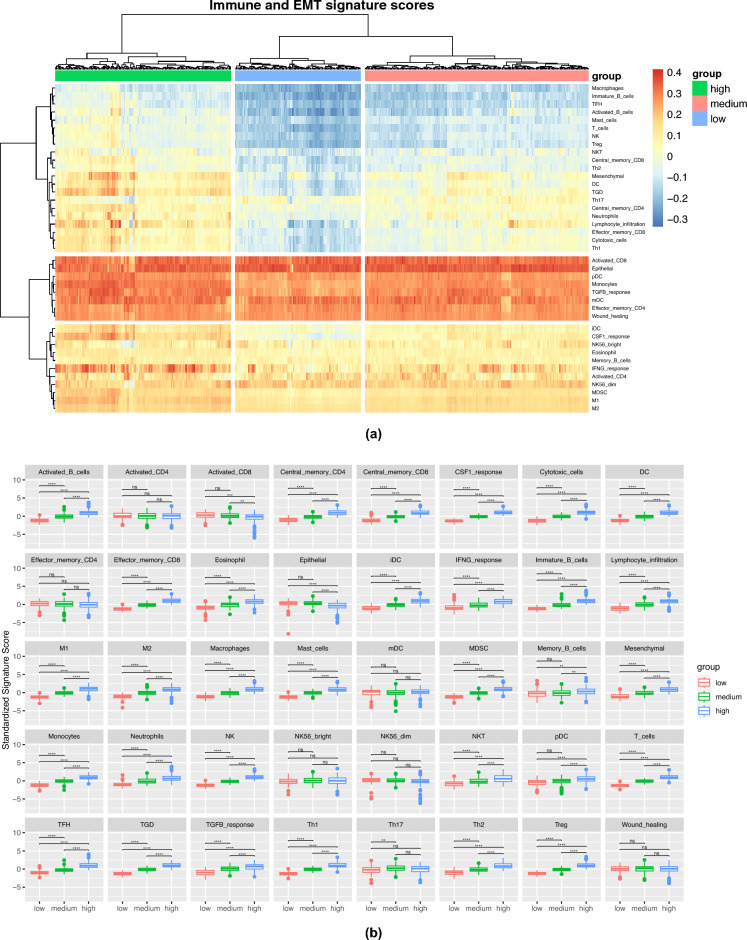
Three clusters of colon cancers have different characteristics of immune cell infiltration. (**a**) The COAD samples were clustered into three groups, namely the low mesenchymal, medium mesenchymal, and high mesenchymal groups, based on their EMT and immune-related gene sets. The heatmap shows the singscore values; each column represents a sample, and each row represents a molecular signature. Positive values indicate gene set enrichment, while negative values indicate inverse enrichment. (**b**) The Student’s t-test comparison of signature scores between each group, with *P*-values adjusted by the Bonferroni correction. ns: adj-$P > 0.05$, *: adj-$P \le 0.05$, **: adj-$P \le 0.01$, ***: adj-$P \le 0.001$, ****: adj-$P \le 0.0001$.

Through the signature score, we identified three clusters characterized by varying levels of mesenchymal activity: high mesenchymal, medium mesenchymal, and low mesenchymal clusters (Fig. 2a). The high mesenchymal cluster demonstrated greater enrichment in various immune cells, such as macrophages, T cells, T-helper 1 (Th1) cells, T-helper 1 (Th2) cells, mast cells, NK cells, NKT cells, and others, except for T-helper 17 (Th17), activated CD4, activated CD8, effector memory CD4, mature dendritic cells (mDC), NK56 bright, and NK56 dim, which were low compared to the medium and low mesenchymal groups (Fig. 2b). We noticed a gradual decrease in the overall infiltration of lymphocytes, macrophages, and monocytes from high mesenchymal to medium mesenchymal to low mesenchymal clusters. To further understand the immune processes associated with each mesenchymal group, we employed five key gene sets representing tumor immune states from Thorsson et al*.*^21^. The high mesenchymal group exhibited significantly higher (*CSF1* response), Interferon-$\gamma$ (*IFNG*) response, and *TGF*-$\beta$ (*TGFB*) response scores than the low mesenchymal group.

### Survival analysis of COAD clusters

**Figure 3:**
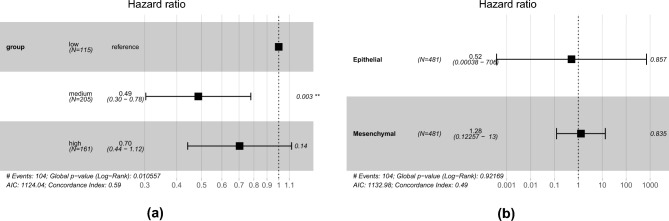
The Cox regression survival analysis of COAD cluster and epithelial-mesenchymal score. (**a**) While no significant distinction exists between patients’ overall status in the high mesenchymal and low mesenchymal groups, the medium mesenchymal group exhibited a significantly poorer survival rate than the low mesenchymal group (hazard ratio $= 0.49$, $P=0.003$). (**b**) We found no significant correlation between overall status and either the epithelial score or the mesenchymal score.

We performed a Cox regression analysis using COAD clusters as covariates to observe their effect on patients’ overall survival. We found no significant difference in overall survival between the high mesenchymal and low mesenchymal groups. Unexpectedly, the medium mesenchymal group had significantly poorer survival than the low mesenchymal group (hazard ratio $= 0.49$, $P=0.003$) (Fig. 3a). Because of this discrepancy, we conducted an additional Cox regression analysis incorporating epithelial and mesenchymal scores as covariates to delve deeper into the details of this discovery. Our findings indicate no significant correlation between overall survival and either epithelial or mesenchymal scores (Fig. 3b).

### Mesenchymal scores were positively correlated with immune cell infiltration scores and higher expression of inflammatory, immunosuppressive, and MHC class 2 immunomodulators

**Figure 4:**
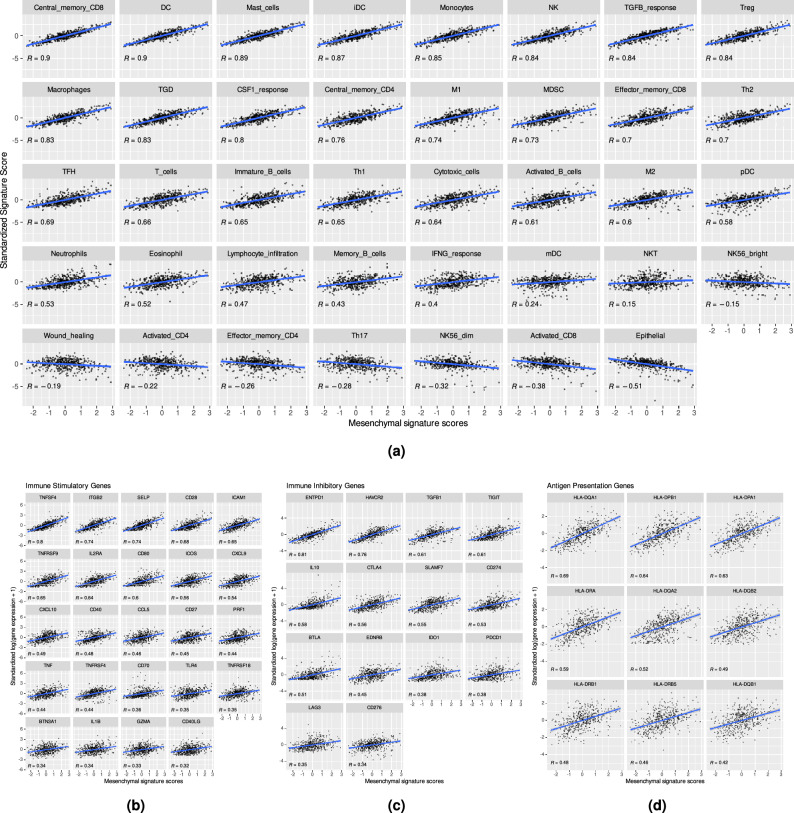
Pearson’s correlation analysis of mesenchymal-immune signature scores and mesenchymal score-immunomodulator gene expression. (**a**) The significant correlations (adj $P < 0.05$) between mesenchymal score and other immune signature scores. (**b–d**) The moderate and strong significant correlations (adj $P < 0.05; |R |> 0.3$) between mesenchymal scores and immunomodulators. The immunomodulators consist of (**b**) immunostimulatory genes, (**c**) immunoinhibitory genes, and (**d**) antigen presentation genes.

Correlation analysis revealed that the mesenchymal states exhibited positive correlations with nearly all signature scores related to immune cells and processes (31 out of 39). However, the anti-tumor cells’ activated CD4 and activated CD8 signatures displayed negative correlations with mesenchymal states (Fig. 4). The central memory CD8, DC, and mast cells displayed the strongest positive correlation with the mesenchymal scores. Interestingly, we also noticed that the immunotolerant immature DC (iDC) had a stronger positive correlation ($R=0.87$) with EMT than mature-activated DC (mDC) ($R=0.24$). Supplementary Table S2 lists all the results of the mesenchymal-immune signature correlation analysis.

Next, we computed the strength of the associations between immunomodulators and EMT states. Fig. 4b–d shows all moderate to strong significant correlations (correlation coefficient, $R >3$; adjusted $P < 0.05$) between immunomodulators and mesenchymal scores. We found that mesenchymal scores were significantly positively correlated with most immunostimulatory genes. Among the stimulatory immunomodulators, *TNFSF4, ITGB2*, and *SELP* showed the highest correlation with mesenchymal scores. Additionally, the receptors of *TNFSF4, TNFRSF4*, and the binding partner of *ITGB2, ICAM1*, had a medium-strong correlation with mesenchymal score ($R = 0.44$ and $R=0.65$, respectively). Similar to the analysis of stimulatory immunomodulators, the mesenchymal scores exhibited moderate to strong positive correlations (adjusted-$P < 0.05$, $R > 0.3$) with all immunoinhibitory genes except for *VEGFA* and *VEGFB*. Among antigen-presentation genes, only MHC class 2 showed a significant moderate-to-strong correlation. In contrast, MHC class 1 genes, such as *HLA-A, HLA-B, HLA-C, MICA*, and *MICB* showed weak or nonsignificant correlations. For detailed results of the correlation analysis between mesenchymal scores and immunomodulators, refer to Supplementary Table S3.

### Differential analysis of genes and identification of important genes using MRF

We identified 4075 DEGs between the normal and tumor samples (Supplementary Table S4). Using these DEGs, we predicted the mesenchymal scores and the scores of five immune expression signatures from Thorsson et al*.*^21^ (e.g., macrophages-monocytes (*CSF1* response), overall lymphocyte infiltration, *TGF-*$\beta$ (*TGFB*) response, *IFN-*$\gamma$ (*IFNG*) response, and wound healing) via multivariate random forest (MRF) with the Mahalanobis distance splitting rule^22,23^. The top 10 important genes for each output are listed in Table 1. The genes that were important for both EMT and the immune process were of particular interest. These genes can be identified by their high mean importance or multiplicative scores. *DOK2, IFI44*, and *WAS* returned the highest mean importance scores. Meanwhile, *DOK2, WAS*, and *SASH3* had the top three score multiplication products. The complete list of genes and their importance scores is presented in Supplementary Table S5.

**Table 1:** The top 10 genes for each output importance score.

Signature outputs	Top 10 important genes
Mesenchymal	*MSRB3, CCDC80, MIR100HG, GLI3, SULF1, COL8A1, DDR2, DOK2, BNC2, LINC01094*
Lymphocyte infiltration	*DOK2, SASH3, WAS, CD27, CD37, IL10RA, MAP4K1, CD79A, HCLS1, APBB1IP*
CSF1 response	*DOK2, SASH3, IL10RA, WAS, HCLS1, MS4A4A, APBB1IP, LINC01094, CD37, CD163*
IFNG response	*IFI44, RSAD2, DOK2, WAS, DDX60, HCLS1, IL10RA, SASH3, LINC01094, FCGR3A*
TGFB response	*MSRB3, GLI3, CCDC80, SULF1, COL8A1, DOK2, DDR2, COL3A1, WAS, MIR100HG*
Wound healing	*MSRB3, MIR100HG, BNC2, CD37, DOK2, IFI44, GLI3, MS4A4A, DDR2, LINC01094*
Mean importance	*DOK2, IFI44, WAS, MSRB3, SASH3, IL10RA, HCLS1, CD37, GLI3, CCDC80*
Multiplicative score	*DOK2, WAS, SASH3, IL10RA, HCLS1, CD37, MS4A4A, LINC01094, APBB1IP, CD163*

## Discussion

EMT has been shown to play a critical role in tumor development and metastasis^5^. The interplay between EMT and the immune microenvironment in cancer is complex and has been investigated in numerous studies^11,24^. As inflammation can induce EMT^11^, we expected to find positive correlations between inflammation markers (such as cytokines and immune cell infiltration) and the colon cancer mesenchymal state. However, as EMT can also modulate the tumor immune microenvironment in colon cancer, we also sought to investigate which routes could contribute to immune escape. As expected, the tumor samples in the high mesenchymal group were characterized by inflammatory TME, with increasing infiltration of both innate (e.g., macrophages, neutrophils, mast cells, and NK cells) and adaptive immune cells (e.g., T cells, cytotoxic T cells, Th1, Th2, and activated B cells). The correlation analysis also showed that most of the immune cell and immunostimulatory cytokines were positively correlated with the mesenchymal score in COAD. In contrast, the low mesenchymal group showed low immune cell infiltration. From the Thorsson gene set, we observed that the high mesenchymal group tended to have higher *CSF1* response, Interferon-$\gamma$ (*IFNG*) response, and *TGF*-$\beta$ (*TGFB*) response scores. Notably, the inflammatory mediator *TGF*-$\beta$ is a principal driver of EMT in cancers^8^. Meanwhile, *CSF1* and *IFNG* play important roles in the breast^25^ and pancreas cancer^26^ EMT processes, respectively.

Special attention should be given to *TGF*-$\beta$ within the Thorsson gene signature. This regulatory factor pivotally shaped the functionality of both adaptive and innate immune cells, including cytotoxic T cells, Th1 and Th2 cells, NK cells, macrophages, and dendritic cells^27^. In cancer, *TGF*-$\beta$ exerts anti-inflammatory effects by facilitating the transition of M1 to M2 macrophages^28^, impeding dendritic cell maturation^29^, suppressing Th1 and cytotoxic T cell responses^30^, promoting Treg cell induction^31,32^, and inhibiting NK cell activity^33^. As an EMT driver, *TGF*-$\beta$ contributes to the activation of both Smad and non-Smad pathways and is mediated by the master regulators of EMT, such as the Snail, Zeb, and Twist proteins^34^. Given its crucial roles in both the cancer immune response and EMT processes, *TGF*-$\beta$ is a vital link bridging the gap between EMT and the tumor immune microenvironment^8^. Therefore, any alterations to *TGF*-$\beta$ will likely affect both the cancer EMT process and the immune response.

Our survival analysis revealed no significant distinction in overall survival between the high mesenchymal group and the low mesenchymal group. Unexpectedly, the medium mesenchymal group exhibited less favorable survival outcomes than the low mesenchymal group. Given these non-linear findings, we conducted an additional Cox regression survival analysis using mesenchymal and epithelial scores to refine our findings. We found no significant correlation between overall status and either mesenchymal or epithelial scores. This result contradicts previous reports^35,36^. However, our results align with Tan et al*.*’s study, which found no correlation between EMT status and overall status for colorectal cancer patients^37^. This could be because EMT status is correlated not only with pro-tumor conditions (TReg, MDSC, CD274) but also with anti-tumor cells (NK cells and M1 cells), as our results show. Recent studies showed that EMT can increase cancer’s vulnerability to NK cell cytotoxicity^38^, and there is a positive correlation between M1 macrophages and EMT^39^. Thus, it is unlikely that EMT status is the sole prognostic factor for survival, as the cellular composition, dedifferentiation grade, and histological subtype may also contribute. For example, Angelova et al*.* demonstrated that immunophenotypes and antigenomic composition can act as prognostic factors in colon cancer^40^, and Ueno et al*.* proposed a prognostic index for colon cancer based on the dedifferentiation grade combined with the histological marker of EMT^41^.

Next, we wanted to know which pathways were involved in EMT-related-immune evasion, as EMT can modulate the tumor immune microenvironment in colon cancer. We found that the infiltration of immunosuppressive cells, such as DC, MDSC^42,43^, Treg^44^, and mast cells^45^, was very strongly correlated ($R > 0.7$) with the mesenchymal score. In the case of DC, iDC had a stronger positive correlation with EMT than mature-activated DC (mDC). Immature DC promoted the immunotolerance of cancer, whereas mature DC effectively promoted an immune response against cancer^46^. The mesenchymal score was negatively correlated with anti-tumor activated CD4 and activated CD8 T cells, indicating that the T cell activation process is altered in tumors with high mesenchymal scores. EMT can inhibit T cell activation by altering antigen presentation^47–49^, expressing immunoinhibitory molecules^26^, or recruiting Treg cells^12,50^. Our results also indicate that immunosuppressive modulators (such as *CD274 (PD-L1), PDCD1 (PD-1), CTLA4, HAVCR2 (TIM-3)*, and *ENTPD1*) play important roles in EMT-associated immune evasion. Our findings support other studies that reported the correlation between immune checkpoints and EMT in various cancers^51–54^. Another immune escape mechanism involves altering the antigen presentation process. An effective anti-tumor immune response requires antigen presentation, in which MHC class 1 molecules, such as *HLA-A, HLA-B*, and *HLA-C*, present antigenic peptides from tumor cells or DC to CD8 T cells. After antigen presentation, CD8 T cells are activated and kill the tumor cells^55^. However, we found only low or nonsignificant correlations between MHC class 1 expression and mesenchymal scores. The transformation of colon cancer cells into mesenchymal phenotypes does not seem to lead to the induction of antigen presentation through MHC class 1. Reduced MHC class 1 antigen presentation by mesenchymal-like cancer cells has also been observed in lung cancers^47^, prostate cancers^48^, and melanoma^49^. The immunosuppressive environment is crucial for mesenchymal-like colon cancer cells to evade immunosurveillance and facilitate metastasis from the colon to other sites. Understanding how EMT influences immune evasion can inform the design and optimization of immunotherapies, potentially making them more effective against a broader range of cancer types. In addition, combining treatments that target both EMT-associated processes and immune evasion mechanisms could offer synergistic effects, potentially enhancing overall treatment efficacy.

The last part of our analysis was to select a relevant subset of genes involved in EMT-immune interactions. To achieve this, we used random forest variable importance measures^56^. To account for multiple outcomes and interdependencies among the outputs, we used the multivariate random forest approach with the Mahalanobis splitting rule^22,23^. Based on the multiplicative scores, *DOK2* was identified as the most important gene for EMT and five intratumoral immune signatures. Docking Protein 2 (*DOK2*) is a member of the *DOK* family and a substrate for many important tyrosine kinases, such as epidermal growth factor receptor, platelet-derived growth factor receptor, and *Her-2*^57–60^. *DOK2* had the highest importance score in the *CSF1* response and lymphocyte infiltration. Some studies report that *DOK2* has crucial functions in immune responses, such as negative regulation of T cell receptor signaling^61^, NK cell activation^62^, and myeloid cell proliferation^63^. According to our findings, *DOK2* was also considered an important predictor of the tumor TGF-$\beta$ response in COAD. We hypothesized that *DOK2* and *TGF*-$\beta$ collaboratively affect the EMT process. Another gene of interest is *MSRB3*. We found that *MSRB3* was the most important predictor of EMT in COAD. *MSRB3* or methionine sulfoxide reductase B3 catalyzes the reduction of methionine sulfoxide to methionine^64^. Recent reports show that *MSRB3* governed EMT and cell stemness via *ZEB1*^65^. We also showed that *MSRB3* is the most important predictor of the *TGF*-$\beta$ response. *DOK2* and *MSRB3*’s roles in the colon cancer immune microenvironment and the EMT process require further investigation, making them interesting subjects for future studies.

Our study could uncover the complex interaction between EMT and the immune process in COAD. A deeper understanding of this interaction can assist researchers in exploring new therapeutic options to enhance colon cancer treatment, particularly by addressing tumor inflammation and metastasis. Furthermore, unraveling the molecular mechanisms responsible for the immunomodulation induced by EMT could reveal new immunomodulatory markers. When combined with the EMT status of tumors, these markers could serve as predictive indicators for both tumor progression and immunotherapies’ effectiveness. Additionally, EMT is a dynamic process, and its effect on immune evasion can vary in different types of cancer and even within different patients. Understanding this variability can help stratify patients based on their specific EMT and immune evasion profiles. Such insights would be a significant stride in oncology, offering valuable information for tailored treatment approaches.

## Methods

### Data acquisition and preparation

We obtained the mRNA expression profiles of COAD (raw counts and transcripts per million (TPM) units) from the GDC-TCGA harmonized database using the Bioconductor package TCGAbiolinks^66^. The database was accessed on January 10, 2023. The dataset included 481 COAD primary tumor samples and 41 normal tissue samples. We filtered out genes with less than five counts across more than 50% of the samples. We also discarded the genes with identical names to enforce unique mapping. Then, we applied a $\log(1+x)$ transformation to the gene expression data in TPM units for transcriptome signature scoring.

### Differential expression analysis (DEA) of the genes

We conducted a differential gene expression analysis using the workflow outlined by Silva et al*.*^67^. This workflow involved using the TCGAbiolinks package to preprocess the data and perform differential expression analysis (DEA). The data preprocessing consisted of three main steps. First, we utilized the TCGAanalyze_Preprocessing function to perform Array Array Intensity correlation and detect outliers. Second, the TCGAanalyze_Normalization function was employed to normalize the mRNA transcripts. The normalization steps in this function consist of within-lane normalization to adjust for the GC-content effect (or other gene-level effects) on read counts and between-lane normalization to adjust for distributional differences between lanes (e.g., the sequencing depth). Finally, we applied the TCGAanalyze_Filtering function to filter out genes with low signals across the samples. After these preprocessing steps, we used the TCGAanalyze_DEA function to identify the DEGs in the normal and tumor samples. We defined the genes with an absolute log fold change of $\ge 1$ and FDR of $\le 0.01$ as significant DEGs.

### Transcriptomic signature scoring and sample clustering

For this study, we used cancer-specific transcriptomic EMT signatures from Tan et al*.*^37^ for both the epithelial and mesenchymal phenotypes of cancer cells. We combined gene sets from Angelova et al*.*^40^, Aran et al*.*^68^, and Thorsson et al*.*^21^ to represent immune cells and the immune process in the tumor microenvironment. Angelova et al*.* developed gene sets that represent various tumor-infiltrating immune cells in colon cancer^40^, while Aran et al*.* provided the M1 and M2 macrophage gene sets^68^. Thorsson et al*.* compiled immune expression signatures from multiple sources and identified five key gene sets for tumor immune states, including lymphocyte infiltration, macrophages and monocytes (the *CSF1* response), the Interferon-$\gamma$ (*IFNG*) response, wound healing, and the *TGF*-$\beta$ (*TGFB*) response^21^. Jaccard similarity was employed to calculate the intersection of gene sets, as described in Maleki et al*.*^20^.

Using the $\log(1+TPM)$ gene expression data for the tumor samples, we applied singscore methods to acquire signature scores for the EMT and immune cells. Then, we employed hierarchical clustering with Ward’s minimum variance method to categorize the samples into subgroups based on the EMT-immune signature scores. To determine the optimal number of clusters, we used the R package NbClust, which consists of 30 indices for assessing cluster numbers.

### Survival analysis

We used univariate Cox regression analysis to analyze the effects of the COAD clusters and epithelial-mesenchymal scores on COAD patients’ overall survival. A *P*-value of $< 0.05$ was considered significant. All survival analyses were performed with the survival and survminer R packages.

### Statistical analysis

To compare the signature score means among different groups, we conducted t-tests between the high mesenchymal and medium mesenchymal groups, the high mesenchymal and low mesenchymal groups, and the low mesenchymal and medium mesenchymal groups. We applied the Bonferroni correction to adjust the *P*-value and considered mean differences significant if the adjusted *P*-value was $< 0.05$.

We standardized each of the signature scores to have zero mean or unit variance. Then, we computed Pearson’s correlation coefficient between the standardized mesenchymal signature score and the immune cell infiltration/process signature scores.

We also investigated the relationships between mesenchymal signature scores and immunomodulator genes from Thorsson et al*.*’s previous study (Supplementary Table S1)^21^. The immunomodulators comprised immunostimulatory, immunoinhibitory, and antigen-presentation (major histocompatibility complex [MHC] class 1 and class 2) genes. We transformed the gene expression data (TPM units) with the $\log(1+x)$ transformation and standardized it to have zero mean or unit variance. Then, we performed Pearson’s correlation analysis between the transformed gene data and standardized mesenchymal scores. We used the Bonferroni correction to adjust the *P*-value and considered correlations significant if the adjusted *P*-value was $< 0.05$.

### Multivariate random forest variable importance

We wanted to find the important genes associated with EMT and intratumoral immune states. To achieve this goal, we used MRF with the Mahalanobis distance splitting rule^22^, which was implemented in the R package randomForestSRC^23^. We trained MRFs with DEGs from previous analyses as inputs to predict the mesenchymal scores and five immune expression signature scores from Thorsson et al*.*^21^. The five immune signatures were lymphocyte infiltration, *CSF1* response, *IFN*-$\gamma$ response, *TGF*-$\beta$ response, and the wound healing process. The expression of DEGs was transformed with a $\log(1+x)$ transformation and standardized to have zero mean or unit variance. The variable importance for each output was determined with the permutation importance method^56^. We calculated the mean importance score and multiplicative score for each gene. The mean importance score was the average of all output importance scores, indicating the effect of a variable or gene on predicting one or more outputs. The multiplicative score was the product of each importance score and represented the gene’s influence in predicting all outputs simultaneously.

### Supplementary Information

Supplementary Table S1.

Supplementary Table S2.

Supplementary Table S3.

Supplementary Table S4.

Supplementary Table S5.

## Data Availability

We downloaded the publicly available COAD dataset from The National Cancer Institute (NCI) Genomic Data Commons (GDC) TCGA https://gdc.cancer.gov/access-data/gdc-data-portal with the TCGAbiolinks package.
